# Can a self-regulated flipped classroom improve creative performance? Evidence from a randomized controlled trial

**DOI:** 10.3389/fpsyg.2026.1807932

**Published:** 2026-05-29

**Authors:** Yongkang Chen, Jinping Feng, Yueqin Ning, Feitian Tang, Liyang Wang, Alireza Mohammadi

**Affiliations:** 1 School of Art and Design, Zhongyuan University of Technology, Zhengzhou, China; 2 Media Arts Research Center, Jiangxi Institute of Fashion Technology, Nanchang, China; 3 Guangzhou Vocational and Technical University of Science and Technology, Guangzhou, China; 4 School of Arts, Nanchang University, Nanchang, China; 5 City Graduate School & Centre of Foundation Studies, City University Malaysia, Petaling Jaya, Malaysia

**Keywords:** art and design education, convergent thinking, creativity, divergent thinking, flipped classroom, self-regulated learning

## Abstract

**Introduction:**

Creativity is widely acknowledged as an essential competency in art and design education, but approaches for developing it through pedagogical interventions have not been sufficiently explored. This study employed a 16-week self-regulated flipped classroom intervention to examine creativity outcomes, focusing on divergent and convergent thinking.

**Methods:**

A randomized controlled trial was conducted with art and design students assigned to either an intervention group (*n* = 24) or a control group (*n* = 24). Creativity was assessed before and after the intervention using the Guilford Alternate Uses Test and the Remote Associates Test.

**Results:**

The findings indicated that students in the flipped classroom performed better on divergent thinking (especially on fluency and originality) than those in traditional classrooms. No significant effects were observed for flexibility, elaboration, or convergent thinking.

**Discussion:**

These findings suggest that self-regulated flipped learning may be an effective approach for fostering generative aspects of creativity in art and design education. This study contributes to the growing literature by highlighting the role of instructional design in supporting different dimensions of creative development.

## Introduction

Because creativity is both the basis of future innovation and a core attribute of art and design learning, it is an essential competency at the core of creativity (Wang et al., 2023), since students have to develop ability to think outside of the box (divergent thinking) and generate products from their thoughts (convergent thinking). Although nurturing creativity is widely recognized as a key educational aim (Benedek et al., 2025), traditional lecture-centric teaching methods prevail in higher education. However, such approaches generally prioritize rote memorization and passive learning over the deep cognitive and metacognitive processes that form the basis of creative development (Kay et al., 2018; Kembara et al., 2019). As a result, considerable research has focused on identifying effective educational techniques for promoting creativity (e.g., Altan and Tan, 2020; Kembara et al., 2019).

The flipped classroom has been recently considered as a remarkable pedagogical innovation that can be beneficial in motivating students' higher-order thinking (Lee and Lai, 2017; Priyaadharshini and Sundaram, 2018). It transfers information acquisition to pre-class activities and utilizes in-class time for active learning and promotes collaboration in the classroom, encouraging students to think critically and thereby improving capacities needed for divergent and convergent thinking (Martinez and Gomez, 2025). Although the overall impact of flipped classrooms is well documented, little research explicitly examines the impact on creativity in art and design education.

Self-regulated learning (SRL) theory (Schunk and Zimmerman, 2012) offers an effective conceptual basis to evaluate how the flipped classroom and other instructional designs can shape students' learning processes. It concerns learners' active control of the cognitive, emotional, and behavioral components of learning in service of educational goals (Boekaerts, 1996). In these settings, students are required to plan, monitor, and reflect during the forethought, performance, and self-reflection stages of learning. These stages align with key instructional aspects of the flipped classroom, such as pre-class preparation, in-class problem solving, and post-task reflection. Although these self-regulatory processes have conceptual parallels with the cognitive demands of creative thought, particularly in planning, idea exploration, and evaluation (Yoon et al., 2021), they are better understood as supportive learning conditions rather than direct drivers of creative performance. In flipped classrooms, activities with students centered in the classroom like brainstorming, problem solving, and peer feedback allow divergent and convergent thinking to occur in an interactive learning environment.

In the flipped classroom model, it can be practically observed that students use videos, books, and some interactive exercises before class to help set learning goals and monitor student understanding before performing activities for pre-class preparation (Han and Klein, 2019; Jensen et al., 2018). This approach challenges students to think broadly and provides them with multiple stimuli, prompting them to develop multiple interpretations and solutions. During the in-class phase, students participate in activities that enhance knowledge transfer, reasoning, and collaborative idea generation, receiving immediate feedback from peers and the instructor (Bosch et al., 2021). This fosters a range of thought processes that lead learners to become more flexible and fluent in their ideas. Convergent thinking, when judgment is assessed with tasks that evaluate and synthesize ideas and make judgments, enables learning to happen more successfully, because these tasks legitimize and enhance students' creative work. So, in the flipped class, this continuous movement of divergent and convergent thinking is maintained through self-directed planning, doing, and reflection.

Despite increasing interest in flipped classroom approaches and creativity in education, critical gaps remain in understanding how they interact. Very few studies have employed rigorous experimental methods such as randomized controlled trials to determine the causal effects of flipped learning on creativity. Therefore, the current study has largely taken a broad look at creativity as a construct, paying relatively little importance to its multidimensionality, especially concerning multiple components of divergent thinking. Therefore, this study examines the cognitive effects of a 16-week SRL-oriented flipped classroom intervention on creativity among art and design students. Accordingly, the following hypothesis is formulated:

H0. There are no differences in improvement across dimensions of divergent thinking (fluency, originality, flexibility, elaboration) and convergent thinking between students in the flipped classroom and those in the traditional classroom.

## Methods

### Participants

An initial pool of 283 students enrolled at the School of Art and Design was considered. To ensure sufficient language proficiency, students who had not passed the College English Test Band 4 (CET-4) were excluded. This resulted in a final eligible student's sample of 54. Further screening criteria included: (1) participation in at least one art or design course during the semester, (2) no prior experience with flipped classroom instruction, and (3) willingness to participate in all course activities and assessments. After applying these criteria, 50 participants were retained.

**Figure 1:**
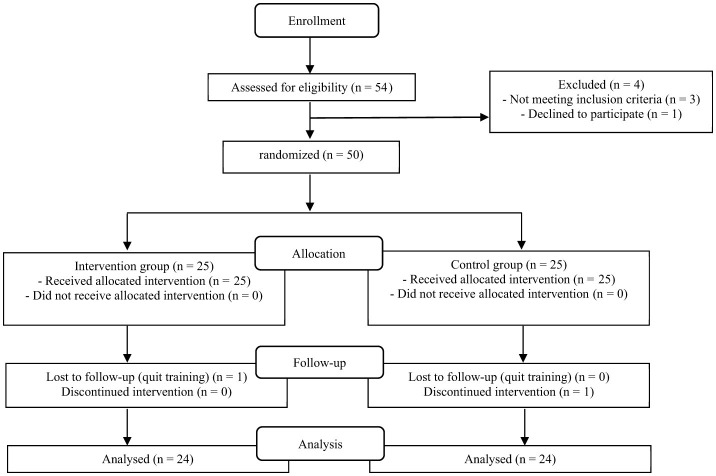
CONSORT flow diagram[124mm][4mm].

A randomized controlled trial was used to examine the effects of the flipped classroom on creativity. Fifty participants were randomly assigned to experimental or control groups using an online randomization tool (https://www.randomizer.org/) with a 1:1 allocation ratio. The experimental group received the flipped instruction, while the control group was taught using traditional methods. Two participants did not complete all sessions and assessments during the intervention and were excluded from the final outcome sampling, which resulted in a total of 48 participants (CONSORT form, Figure 1). A *post hoc* power analysis was performed using G^*^Power 3.1 (Heinrich Heine University Düsseldorf, Germany) for a repeated-measures ANOVA with a within-between interaction. The achieved power was 0.924, with alpha set at 0.05, a medium effect size (*f* = 0.25), and a total sample size of 48. This study was approved by City University Malaysia Research Ethics Committee (CITYU/RMC/R.ETHICS 2822) on 19 March 2025 and conducted in accordance with the Declaration of Helsinki.

### Measurements

#### Convergent thinking

Convergent thinking was measured using the remote associates test (RAT), first devised by Mednick (1962). Previous studies have found that this test has strong internal consistency (Cronbach's alpha = 0.85–0.87) (Chermahini et al., 2012; Colzato et al., 2013) confirming its reliability. In the RAT, participants were asked to identify one word that links three unrelated words. For example, the answer for the triad “Ship/Stick/Back” is “yard” as it forms compounds such as “shipyard,” “yardstick,” and “backyard.” The answer must form valid compounds; descriptive responses such as “Big Stick” or “Beautiful Ship” are not acceptable. The inter-rater reliability for convergent thinking was good (ICC = 0.871).

#### Divergent thinking

The Guilford alternate uses test (GAU) (Guilford, 1967) was used to evaluate divergent thinking. The reliability of this test has been demonstrated in numerous studies, with Cronbach's alpha scores of 0.85 for flexibility, 1.00 for fluency, and 0.71 for originality (Martinková et al., 2022). The GAU has been widely used to assess interventions intended to foster creativity (Hocevar, 1980; Neville and Makopoulou, 2020). It is particularly valued because it does not exhibit short-term learning effects, allowing it to be applied in pre- to post-intervention testing (Oppezzo and Schwartz, 2014).

Participants were asked to consider two common objects, such as a toothbrush or a brick, and generate as many new and useful ways to use them as possible within 8 min (4 min for each object). For example, when asked about potential uses for a pen, participants proposed ideas such as “transforming the pen cap into doll legs,” “utilizing the cap as a whistle,” or “employing the tube as a snorkel.” To avoid learning effects, the objects were changed to be a newspaper, a plastic bottle and an electric wire in the post-test.

Responses were assessed by two trained independent raters who were blinded to group assignment. The raters were calibrated using a pilot dataset to ensure scoring consistency. An idea was considered fluent if it was feasible, distinct, and clearly articulated. “Create a gun barrel from a pen tube,” for instance, would be excluded as it was not feasible. Responses that were too general or repetitive, such as “use the pen for carrying,” were excluded unless they demonstrated sufficient specificity. For flexibility scoring, responses under the same conceptual category (e.g., “store water in the pen tube” and “carry oil in the pen tube”) were combined and assigned a single flexibility score. Originality scores were assigned to ideas that were novel and not repeated by more than 5% of the participants. The inter-rater reliability for fluency (ICC = 0.779), flexibility (ICC = 0.814), originality (ICC = 0.758), and Elaboration (ICC = 0.952) remained consistently high across both pre- and post-tests.

#### Self-reflection survey

The survey was aimed at measuring students' beliefs around their experience in the flipped classroom, with regard to SRL (Schunk and Zimmerman, 2012) and creativity development. The survey consisted of Likert scale items that addressed several key areas of the students' learning experience, namely engagement; perceived control of learning, creativity development; interaction in class. The questionnaire was administered at the post-test to capture students' perceptions of their learning experience following the intervention. The items consist of four domains; they are engagement and learning control, creativity development, class interaction and collaboration within the classroom context.

### Procedure

The flipped classroom intervention was implemented over a 16-week period. Students were required to complete preparatory materials before class, while in-class time was devoted to interactive and creative activities designed to promote engagement and higher-order thinking. Creative performance (divergent and convergent thinking) was assessed at both pre-test and post-test, while the SRL survey was administered at the post-test to capture students' perceptions of their learning experience following the intervention. The GAU and RAT tasks were carried out in English. Instructions were provided in Chinese, and practice examples were given before formal testing to ensure participants' understanding.

Pre-class materials included instructional videos, readings, and guided exercises, while in-class activities included brainstorming tasks (e.g., generating unusual uses for everyday objects), analysis of surrealist artworks (e.g., Salvador Dalí and René Magritte), and collaborative design projects. Students completed practical exercises using digital tools (e.g., Photoshop), with peer discussion and feedback to aid idea development and refinement. As the course progressed, students engaged in more complex tasks requiring them to apply creative thinking and technical skills, culminating in final project presentations and reflective discussions. Throughout the intervention, the instructor served mainly as a facilitator, guiding discussions and providing feedback rather than delivering traditional lecture-based instruction.

### Statistical analysis

A two-way repeated measures ANOVA was conducted to measure the impact on creativity. This analysis assessed the interaction between time (pre-test vs. post-test) and group (intervention vs. control) on divergent and convergent thinking. Normality was assessed using the skewness and kurtosis indices, and homogeneity of variance was assessed using Levene's test. The independent samples *t*-test was applied to compare the survey responses for the two groups. Finally, Spearman's rank correlation was used for examining relationships between survey responses and scores on the GAU and RAT to determine the extent to which students' self-reported learning experiences were related to their creativity scores. All statistical analyses were performed using SPSS 28.0 (IBM Corp., USA) at an alpha level of 0.05.

## Results

Normality was assessed using skewness and kurtosis indices, and all variables fell within acceptable ranges (skewness < |2| and kurtosis < |7|), indicating no significant deviation from normality (West et al., 1995). Moreover, homogeneity of variance was tested using Levene's test, and the assumption was satisfied (all *p* > 0.05).

Results indicated that the flipped classroom intervention had significant effects on several creativity dimensions (Table 1). For fluency, the test effect was significant (*F* = 68.856, *p* < 0.001, $\eta_{p}^{2}$ = 0.599), and the interaction effect was also significant (*F* = 14.609, *p* < 0.001, $\eta_{p}^{2}$ = 0.241). For originality, the test effect was significant (*F* = 24.430, *p* < 0.001, $\eta_{p}^{2}$ = 0.347) and the interaction effect was also significant (*F* = 10.136, *p* = 0.003, $\eta_{p}^{2}$ = 0.181). For flexibility (*F* = 164.662, *p* < 0.001, $\eta_{p}^{2}$ = 0.782) and elaboration (*F* = 4.694, *p* = 0.035, $\eta_{p}^{2}$ = 0.093), the test effect was significant; however, the group and interaction effects were not significant.

**Table 1:** Effects of the intervention on creativity dimensions.

Variable	Intervention	Control	Effects	*F*	*p*	$\eta_{p}^{2}$
	Mean ±SD	Mean ±SD				
Fluency						
Pretest	3.864 ± 0.089	3.864 ± 0.147	Test	68.856	< 0.001^**^	0.599
Posttest	3.991 ± 0.107	3.911 ± 0.146	Group	1.367	0.248	0.029
			Interaction	14.609	< 0.001^**^	0.241
Originality						
Pretest	4.893 ± 0.119	4.881 ± 0.090	Test	24.430	< 0.001^**^	0.347
Posttest	4.989 ± 0.132	4.902 ± 0.095	Group	2.847	0.098	0.058
			Interaction	10.136	0.003^*^	0.181
Flexibility						
Pretest	3.130 ± 0.635	3.241 ± 0.689	Test	164.662	< 0.001^**^	0.782
Posttest	4.402 ± 0.059	4.396 ± 0.055	Group	0.290	0.593	0.006
			Interaction	0.375	0.543	0.008
Elaboration						
Pretest	4.626 ± 0.224	4.629 ± 0.234	Test	4.694	0.035	0.093
Posttest	4.671 ± 0.197	4.643 ± 0.253	Group	0.040	0.842	0.001
			Interaction	1.383	0.264	0.029
Convergent thinking						
Pretest	3.645 ± 0.087	3.633 ± 0.092	Test	0.284	0.596	0.006
Posttest	3.646 ± 0.076	3.641 ± 0.094	Group	0.135	0.715	0.003
			Interaction	0.190	0.665	0.004

^*^p < 0.05, ^**^p < 0.001.

The Bonferroni *post-hoc* test results (Table 2) further illustrate the effects of the intervention across creativity dimensions. Both the intervention [MD = 0.127, 95% CI (0.098, 0.157), *p* < 0.001] and control group [MD = 0.047, 95% CI (0.017, 0.077), *p* = 0.003] showed significant improvements from pre- to post-test for fluency, with the between-group difference being significant at post-test (*p* = 0.034). For originality, only the intervention group showed significant improvement over time [MD = 0.096, 95% CI (0.063, 0.130), *p* < 0.001] and a significant post-test difference favoring the intervention group (*p* = 0.011). For flexibility and elaboration, within-group comparisons revealed significant improvements in the intervention group [flexibility: MD = 1.271, 95% CI (1.002, 1.540), *p* < 0.001; elaboration: MD = 0.045, 95% CI (0.007, 0.083), *p* = 0.022], but no significant post-test differences were found between groups (*p* > 0.05). No significant differences were found for convergent thinking between or within groups [intervention: MD = 0.001, 95% CI (−0.024, 0.025), *p* = 0.946; control: MD = 0.008, 95% CI (−0.016, 0.033), *p* = 0.496].

**Table 2:** Results of pairwise comparison using Bonferroni *post hoc* test.

Variable	Group	*N*	MD (SE)	95% CI		Comparison within group	Comparison between group (post-test)
				LB	UB	*p*	*p*
Fluency	Intervention	24	0.127 (0.015)	0.098	0.157	< 0.001	0.034^*^
	Control	24	0.047 (0.015)	0.017	0.077	0.003	
Originality	Intervention	24	0.096 (0.017)	0.063	0.130	< 0.001	0.011^*^
	Control	24	0.021 (0.017)	−0.013	0.055	0.220	
Flexibility	Intervention	24	1.271 (0.134)	1.002	1.540	< 0.001	0.745
	Control	24	1.155 (0.134)	0.886	1.425	< 0.001	
Elaboration	Intervention	24	0.045 (0.019)	0.007	0.083	0.022	0.662
	Control	24	0.013 (0.019)	−0.025	0.052	0.487	
Convergent thinking	Intervention	24	0.001 (0.012)	−0.024	0.025	0.946	0.841
	Control	24	0.008 (0.012)	−0.016	0.033	0.496	

^*^p < 0.05; MD, mean difference (post–pre); SE, standard error; LB, lower bound; UB, upper bound; comparison between group: post-test between intervention and control groups.

In addition, an independent-samples *t*-test was performed to compare SRL scores. Results indicated that the intervention group (*M* = 3.79 ± 1.06) scored significantly higher than the control group (*M* = 2.00 ± 1.02), *t*_(46)_ = 5.956, *p* < 0.001, mean difference = 1.79, 95% CI [1.186, 2.397], Cohen's *d* = 1.723. However, the correlation analysis (Table 3) indicates that SRL was not significantly correlated with creativity.

**Table 3:** Correlations between creativity and self-regulated learning.

Variable	Fluency	Originality	Flexibility	Elaboration	Convergent_T	SRL
Fluency	1					
Originality	0.076	1				
Flexibility	−0.341^*^	−0.153	1			
Elaboration	0.267	0.12	−0.097	1		
Convergent_T	−0.023	−0.195	−0.213	0.098	1	
SRL	0.014	0.104	0.06	−0.037	−0.034	1

Convergent_T, convergent_thinking; SRL, self-regulated learning. ^*^p < 0.05.

## Discussion

### Effects of flipped class teaching on divergent thinking

This work shows that students' divergent thinking, especially as measured through fluency and originality, was significantly improved in the flipped classroom. In the context of art and design education, these improvements are particularly meaningful because fluency and originality represent a core capacity required for exploring unconventional solutions and sustaining effective concept development in studio-based learning. These results align with instructional theories highlighting the role of active and learner-centered environments in promoting creative engagement. Instead of attributing the increased creativity observed in the end result to SRL as a direct mechanism, a flipped classroom also promotes creativity by redirecting instructional time into higher-order cognitive tasks (e.g., ideation, exploration, and in-class application). By transitioning to pre-class content acquisition, students were afforded larger exposure during class to creative tasks, the consideration of alternative perspectives, and incremental refinement of thought processes that lie at the heart of divergent thinking. This reallocation is particularly significant in art and design education, which tends to develop creativity through doing, testing, revising, and dialogue rather than through passive information absorption. These results suggest that improvements in creativity may be more closely associated with task-level instructional affordances and in-class cognitive engagement than with general self-regulatory capacities.

In traditional lecture-based classrooms, learning can be sufficiently passive to provide fewer opportunities for creativity. The traditional lecture environment, in which students acquire knowledge passively, may limit the creativity they experience (Kay et al., 2018). In contrast, the intervention promotes autonomy, as students' complete coursework before class and then generate ideas through discussions and group activities (Fathi and Rahimi, 2020; Holloway et al., 2024; Mustapha and Abdullah, 2024). Structured pre-class presentation of topics likely helped students consolidate and reorganize their ideas, resulting in greater ideational fluency (Han and Klein, 2019; Jensen et al., 2018). For art and design students, such gains in fluency are educationally important because the ability to produce a broad range of ideas is essential during the early stages of brainstorming, sketching, and conceptual exploration, where quantity often provides the basis for later refinement and innovation.

Interactive exercises allow students to receive feedback as they proceed and interact with one another, which may have helped them generate more original ideas and arrive at more relevant solutions (Negrón et al., 2025). This may be because generating novel ideas often involves moving beyond conventional solutions, and reliance on passive learning strategies may limit opportunities for creative engagement (Gamo, 2022; Roberts, 2019). By encouraging experimentation and risk-taking in problem solving, the flipped model helped students move beyond their comfort zones and apply what they learned in problem-solving situations. Furthermore, in-class group activities provided opportunities for divergent conversations in which peer perspectives may have sparked new ideational pathways. This echoes earlier research on cognitive flexibility, which found positive effects of active learning environments on originality through increasing cognitive flexibility and preventing conventional concepts from becoming fixed (Hao et al., 2020; Sawyer, 2011). Moreover, creative learning in art and design contexts is inherently embodied, experiential, and socially mediated. Participatory and arts-based pedagogies focus on active engagement with materials, iterative experimentation, and collaborative meaning-making (Eisner, 2003; Moreno et al., 2023). In this light, the observed improvements in fluency and originality may be attributed to both increased cognitive engagement and the richer experiential and socially situated learning environments fostered by the flipped classroom (Al-Zahrani, 2015).

The flipped classroom had no significant impact on flexibility compared with the control group. An alternative explanation is that students improved the fluency (number of ideas developed) and originality (innovative solutions) but not in how to pass from one domain to another, where it is possible that cognitive flexibility is key. This type of flexibility involves shifting between perspectives and reframing problems in creative ways, which may require more formal instruction or targeted training in cognitive reframing strategies (Weatherford et al., 2020). Such flexibility is particularly important in art and design education, where students must reinterpret design briefs, draw on cross-domain inspiration, or shift between functional, aesthetic, and user-centered approaches.Future interventions to improve flexibility might include concept mapping, analogical reasoning exercises, or cross-disciplinary problem-solving tasks within the flipped classroom approach.

In general, participants in the experimental group reported significantly higher levels of SRL than those in the control group at post-test, reflecting differences in self-reported learning experiences between conditions. In the current study, however, SRL was not significantly associated with any dimension of creative performance, suggesting it may not serve as a direct pathway for enhancing creativity. This means that students' perceived learning experiences did not correlate with objective measures of creative performance. One possible reason is that SRL is more representative of a general learning orientation, while creativity, particularly divergent thinking, is more strongly influenced by task-specific cognitive processes such as in-class cognitive engagement and interactive idea generation. Indeed, SRL may be better understood as a parallel outcome reflecting the general effects of the flipped classroom on students' engagement and learning orientation, rather than as a direct influence on creativity. Subsequent investigations on the specific mechanisms from flipped classrooms that influence the increase in creative performance, for example in-class cognitive engagement (Wang et al., 2026) and momentary motivational states (Karimi et al., 2022), should be emphasized.

### Effects of flipped class teaching on convergent thinking

Conversely, there was no significant intervention effect on convergent thinking, as indicated by the non-significant time-by-group interaction. This finding suggests that flipped learning may promote idea generation (divergent thinking) but does not itself enhance students' ability to synthesize and select among competing solutions (Rahayu et al., 2025).

Notably, a thinking modality related to this is convergent thinking, which is the ability to critically process, polish, and integrate information to a single right or best solution (Lee et al., 2025). Unlike divergent thinking, which thrives in fluid, exploratory environments, convergent thinking relies more on structured reasoning, logical deduction, and precision. The flipped classroom model promotes collaborative problem-solving and critical discussion, yet these activities are predominantly focused on the initial, open-ended stages of idea generation, not the analytical evaluation and selection of the best potential outcome (Padugupati et al., 2021; Rahayu et al., 2025). As a result, students may not have received sufficient guidance and practice in analytical decision-making, resulting in no observable improvements in convergent thinking.

This finding may also be explained by the nature of art and design education. Creative disciplines tend to value multiple acceptable outcomes over a single correct answer (Bleiker, 2021), which contrasts with the rigid evaluative demands of convergent tasks such as the RAT used in this study.The flexibility of the flipped classroom structure and the ability to explore freely may have failed to set up the limitations and evaluative checks needed to channel convergent reasoning (Cheng et al., 2018). Future studies may investigate whether convergent thinking skills can be strengthened by integrating structured critical analysis exercises, decision-making tasks, or guided critique sessions into the flipped model.

Furthermore, this study established no significant correlation between SRL and convergent thinking. Unlike its potential association with fluency and originality, self-regulation was not predictive of evaluative problem-solving ability. In other words, there is little apparent correlation between goal setting, self-monitoring, and reflective learning practices and whether or not goal setting, self-monitoring, and reflective learning practices appear to have little bearing on convergent reasoning processes, even though they may support the exploratory nature of divergent thinking. This is consistent with previous findings suggesting that convergent thinking relies more on organized knowledge retrieval, deductive reasoning, and domain-specific experience (Eglington and Kang, 2018) than on self-regulation alone.

## Limitation and recommendation for future study

Several limitations of this study should be acknowledged. First, the creativity measures employed may not fully capture the broad and multidimensional nature of creativity. Although the GAU and RAT are widely used and validated measures of divergent and convergent thinking, they primarily assess specific cognitive components rather than creativity in its entirety. As creativity in art and design often involves visual, spatial, and experiential processes, these measures may not fully reflect real-world creative problem solving. This concern aligns with earlier research indicating that performance on divergent and convergent thinking tasks does not necessarily translate to applied creative performance (Baer, 2011; Weisberg, 2023). More comprehensive creativity assessment strategies would benefit future research. Alternative measures (e.g., portfolio-based assessments, peer-reviewed design projects, or authentic creative tasks) might offer more ecologically valid assessments of creative performance (Christiaans, 2002). The EPoC battery (Lubart et al., 2019) and creative self-efficacy (Karwowski et al., 2018; Shaw et al., 2021) and creative self-regulation (Ivcevic et al., 2024) as recent frameworks for such analysis may further enrich our understanding of creative development. These approaches might further clarify the link between subjective learning experiences and the objective performance outcomes observed in this study. Future studies could also investigate the long-term sustainability of creativity gains through flipped classroom practice.

Second, several methodological considerations should be noted when interpreting the data. This study was conducted at a single institution, which may limit the generalizability of the findings to other educational settings, institutions, or student populations. Additionally, as a single instructor was involved, teaching style and interaction patterns may have influenced the results. Even with controlled conditions, some degree of informal student interaction between groups cannot be entirely excluded. Future studies could be strengthened by including multiple institutions and instructors, and by implementing stricter controls on group interactions to improve the credibility and generalizability of the results.

## Conclusion

This study indicated that flipped learning may foster divergent thinking in art and design students, with stronger effects on fluency and originality than on flexibility, elaboration, or convergent thinking. The flipped classroom appears to be a promising environment for stimulating the generative components of creativity through student-led ideation. Nonetheless, the evaluative and integrative aspects of creative work may be better served by more explicit guidance on analytical approaches and structured scaffolding. These results underscore the need to align instructional design with the cognitive requirements of different types of creative activity. From this perspective, flipped classrooms represent a productive pedagogical approach for developing creativity when accompanied by targeted instructional design.

## Data Availability

The datasets presented in this study can be found in online repositories. The names of the repository/repositories and accession number(s) can be found in the article/Supplementary material.
